# The lysosome-related characteristics affects the prognosis and tumor microenvironment of lung adenocarcinoma

**DOI:** 10.3389/fmed.2024.1497312

**Published:** 2025-01-07

**Authors:** Wuguang Chang, Wuyou Gao, Yawei Wu, Bin Luo, Lekai Zhong, Leqi Zhong, Wenqian Lin, Zhesheng Wen, Youfang Chen

**Affiliations:** ^1^Guangdong Provincial Key Laboratory of Biomedical Imaging and Guangdong Provincial Engineering Research Center of Molecular Imaging, The Fifth Affiliated Hospital, Sun Yat-sen University, Zhuhai, China; ^2^Department of Thoracic Surgery, State Key Laboratory of Oncology in South China, Collaborative Innovation Center for Cancer Medicine, Sun Yat-sen University Cancer Center, Guangzhou, China; ^3^Department of Anesthesiology, State Key Laboratory of Oncology in Southern China, Collaborative Innovation Center for Cancer Medicine, Sun Yat-Sen University Cancer Center, Guangzhou, China; ^4^School of Laboratory Medicine and Life Sciences, Wenzhou Medical University, Wenzhou, China

**Keywords:** lysosome, prognostic model, CTSH, lung adenocarcinoma, immunotherapy

## Abstract

**Background:**

The lysosome plays a vitally crucial role in tumor development and is a major participant in the cell death process, involving aberrant functional and structural changes. However, there are few studies on lysosome-associated genes (LAGs) in lung adenocarcinoma (LUAD).

**Methods:**

Bulk RNA-seq of LUAD was downloaded from The Cancer Genome Atlas (TCGA) and Gene Expression Omnibus (GEO). The lysosome risk signature was constructed after univariate and least absolute shrinkage and selection operator (Lasso) cox regression analysis of the TCGA training set, and its capability was validated by additional validation sets from GEO. Single cell sequencing (scRNA) was obtained from GEO to analyze the differences of lysosome risk signature at the single-cell level and the differences in the function and pathway. In vitro experiments have validated the function of CTSH in LUAD.

**Results:**

The risk signature contained seven key LAGs, and patients were categorized into high- and low-risk groups based on a specific calculation formula. The LAG risk signature, which accurately predicted the prognostic status of LUAD patients, was still regarded as an independent prognostic indicator in multifactorial cox regression analysis. Subsequently, the combination of the signature and key clinical information was used to construct a column-line diagram for clinical assessment, which had a high discriminatory power. Immune infiltration analysis from bulk RNA-seq and scRNA-seq indicated that the low-risk group was immune-activated and had a better benefit in the prediction of immunotherapy. Finally, we validated its role in inhibiting tumor proliferation and metastasis in LUAD cells by knockdown of CTSH.

**Conclusion:**

We defined a new biomarker that provided unique insights for individualized survival prediction and immunotherapy recommendations for LUAD patients.

## 1 Introduction

Non-small cell lung cancer (NSCLC) is the most common-sense subtype of lung cancer worldwide, and its epidemiology and current state of treatment are evolving (1). According to the latest epidemiological data, NSCLC ranks first in the world in terms of morbidity and mortality (2), which may be partly attributed to factors such as smoking, environmental pollution, and population aging (3). In the meanwhile, advances in genomics and molecular biology have revealed the molecular diversity of NSCLC, providing more possibilities for individualized treatment (4).

The treatment of non-small cell lung cancer is becoming more and more diversified. In addition to traditional surgery, radiotherapy and chemotherapy, novel therapies such as targeted therapy and immunotherapy have been gradually applied in the clinic (5). Immune checkpoint inhibitors (e.g., PD-1/PD-L1 and CTLA-4 inhibitors) and targeted therapies (e.g., EGFR inhibitors, ALK inhibitors) have become an integrated part of NSCLC treatment (6). The individualization of treatment plans is also more dependent on the molecular characteristics of the lung cancer, as well as the patient’s genetic background and physical condition. While novel treatments offer hope, they are also accompanied by a series of challenges. These include drug resistance, the high cost of treatment, and individual differences in clinical practice (7). Hence, the treatment of NSCLC continues to require interdisciplinary collaboration and sustained research efforts to improve patient survival and quality of life.

The lysosome contains a large number of protein hydrolyzing enzymes and in the past were known to be one of the key digestive organelles for maintaining homeostasis in the organism (8). With the in-depth study of lysosomal function, researchers have found that it plays a key role in tumors, regulating the balance of apoptosis and autophagy, invasion, metastasis, drug resistance, and immune response, and affecting tumor biology and therapeutic response (9, 10). Tumors can regulate their own metabolism by altering the number of lysosomes, the activity and expression of proteins, and their spatial distribution, which in turn promotes progression (11). Activation of the autophagy-lysosomal pathway is regarded as a cytoprotective response to anticancer drug therapy and enhances tumor resistance to radiotherapy, chemotherapy, and targeted therapy (12). Targeting lysosomes is instrumental in preventing or delaying tumor drug resistance. In addition, lysosomes are major regulators of apoptosis, autophagy, necrosis, and other modes of cell death (13). Targeting aberrantly activated lysosomes in tumors to promote tumor death is a tenable therapeutic strategy.

In this study, we constructed a prognostic model for LUAD using LAGs, which was well capable of predicting the prognostic status of LUAD patients and was validated in extra datasets. In addition, we found that CTSH, a key gene in lysosome, inhibited the progression of LUAD by *in vitro* experiments, which is significantly valuable for the development of targeted lysosomal therapies.

## 2 Materials and methods

### 2.1 Data acquisition and processing

RNA-seq for the training set was obtained from the TCGA database, and a total of 485 LUAD patients were collected after deleting duplicate sequencing data, incomplete survival information, and samples with a survival time of less than 30 days. The validation sets GSE72094 and GSE68465 were obtained from GEO, which contain 398 and 442 samples with complete clinical information, respectively. LAGs were downloaded from MSigDB (KEGG_LYSOSOME) (14), containing a total of 121 genes (Table S1).

### 2.2 Identification of key lysosome-associated genes

Firstly, the differentially expressed genes between tumor and normal tissues in TCGA-LUAD were compared by limma difference analysis (*p* < 0.05, fold change ≥1) (15), and key LAGs were obtained by analyzing and comparing KEGG_LYSOSOME.

### 2.3 GO/KEGG enrichment analysis

Potential pathways involved in key LAGs were analyzed by GO/KEGG enrichment, a procedure executed by the “clusterProfiler” package (16).

### 2.4 Construction of lysosome risk signature

Univariate cox regression analysis was applied to key LAGs, which were later included in lasso cox regression analysis to identify candidate genes and their coefficients. The coefficients of each gene were multiplied by its RNA expression, which was finally summed up to be the risk scores of each patient. The LUAD patients were divided into high- or low-risk groups according to the median of their risk scores and subjected to subsequent analysis to compare the survival differences between the two groups by KM curves and log-rank tests. Risk scores were calculated in the same way for two validation sets.

### 2.5 Establishment and validation of nomogram

In an attempt to improve application of the signature, the lysosome risk signature and clinical characteristics were integrated and a nomogram was constructed by “regplot” R package. ROC curves as well as calibration curves were used to evaluate the predictive performance of the nomogram.

### 2.6 Gene set enrichment analysis

For exploring the biological characteristics and pathways associated with various risk subgroups, we downloaded “c5.go.v7.5.1.symbols” and “c2.cp.kegg.v7.5.1.symbols” from the molecular signature database to be used for implementation in GSEA (17).

### 2.7 Tumor mutation burden analysis

Somatic mutation data of LUAD patients were retrieved from the TCGA database, and the TMB of each sample was obtained by quantifying the number of somatic non-synonymous mutations in the characterized genomic regions. The somatic mutations in the high and low-risk groups were visualized using the “maftools” R package, and the relationship between the risk score and TMB was calculated through correlation analysis.

### 2.8 Immune infiltration analysis and prediction of immune therapy response

The ssGSEA algorithm was used to assess the relative activity levels of 28 immune cells in each sample (18). The ESTIMATE algorithm uses the expression profiles of immune-related genes as well as mesenchymal-related genes to infer the proportion of immune and mesenchymal cells in a tumor, which in turn yields an immune score and mesenchymal score for each sample (19). The Tumor Immune Dysfunction and Exclusion (TIDE) algorithm inferred the response of solid tumors to treatment with immune checkpoint inhibitors (ICIs) by analyzing the degree of immune cell infiltration as well as immune dysfunction (20), and calculated a TIDE score for each patient based on the expression profile of the LUAD, with higher TIDE scores implying a greater likelihood of ICIs treatment Resistance.

### 2.9 The analysis of scRNA-seq

Single cell dataset of LUAD was downloaded from previous research (21)containing 10 LUAD and 10 adjacent normal lung tissues. Processing and analysis of scRNA-seq using “Seurat” (22). Firstly, genes expressed in less than 3 cells were removed and the number of genes expressed in each cell was limited to 500–5,000. Subsequently, cells with a mitochondrial gene proportion exceeding 15% were filtered, and the total number of molecules detected within the cells was less than 40,000. The annotation of cells was based on recognized typical biomarkers. The LAG risk signature was defined as 7 genes in the prognostic model (AP1S1, CTSG, CTSH, CTSV, CTSW, DNASE2B, and NAPSA). The ‘AddModulusScore’ function was used to calculate the LAG risk signature for each cell.

### 2.10 Cellchat analysis of different LAG risk group

Based on the CellChat database of 1939 validated molecular interactions, we simulated the probability of cell–cell communication between different cell types, and inferred the communication between different cell subpopulations (23).

### 2.11 Cell culture

Two human lung cell lines (A549 and PC9) were purchased from the Xinyuan Biotech Co. Ltd. (Shanghai, China) and applied in the experiment. Short tandem repeat (STR), bacterial, mycoplasma and fungal contamination analysis were checked routinely. Cultured in RPMI-1640 medium (Gibco, Grand Island, NY, United States) containing 10% foetal bovine serum (Sinsage, Beijing, China), the cells were cultured in a 5% CO2 humidified environment at 37°C. The siRNAs were from commercial synthesis and transfected into A549 and PC9 cells with GP-transfect-Mate (Suzhou Gene Pharma, China). The sequences of the siRNAs were as follows: si1-CTSH: 5′-CAAGTCATGGATGTCTAAGCACC-3′; si2-CTSH: 5′-CATTGT TGTGGGCGTTTATCTTC-3′; si-NC: 5′-UUCUCCGAACGUGUCA CGUTT-3′.

### 2.12 Western blotting

Protein lysates were prepared with RIPA lysis buffer supplemented with protease inhibitors and phosphatase inhibitors (purchased from Sigma-Aldrich). Samples were quantified by the Bradford method and equal amounts of proteins were separated on 10% Bis-Tris gels (Epizyme, Shanghai, China) using MOPS/MES buffer. The electrodepositionally separated proteins were then transferred onto a polyvinylidene difluoride (PVDF) membrane (Invitrogen, California, United States). Membranes were blocked with 5% skimmed milk (dissolved in PBS containing 0.1% Tween 20) for 2 h at room temperature and then incubated in primary antibodies: mouse anti-TUBLIN (Proteintech, Chicago, USA) 1:3000 and rabbit anti-CTSH (Abcam, Shanghai, China) 1:1000 overnight at 4°C. The following day, the membranes were subjected to incubation with horseradish peroxidase (HRP)-conjugated secondary antibody diluted with PBST containing 5% skimmed milk at room temperature for 1 h. Protein signals were detected using an ECL chemiluminescence system (Tannen, Shanghai, China) and analyzed by Image J software (version 2.0, LOCI, University of Wisconsin, Madison, WI, United States).

### 2.13 Viability and proliferation testing

Assessment of cell viability was done using Cell Counting Kit-8 (CCK-8) (DOJINDO). Cells were cultured in 96-well plates (1,000 cells per well, 100ul complete medium). After affixing, 10ul CCK-8 solution was added to each well and incubation for 2 h, absorbance was measured automatically at 450 nm using a microplate reader (Infinite F50, Tecan Group Ltd., Mannedorf, Switzerland). The assay was performed continuously for 6 days. The proliferation ability of the cells was detected according to the EDU datasheet (Epizyme, Shanghai, China). Proliferation rate of cells = EdU-positive cells/Hoechst-stained cells × 100%.

### 2.14 Colony formation experiments

Cells were seeded in 6-well plates with 1,000 cells per well. Follow up with regular observations and liquid changes. When the number of cells in a single clone was observed to be greater than 50 under the microscope, the culture was stopped. The cells were washed by PBS for 3 times, fixed by 4% paraformaldehyde for 30 min, washing by PBS for 3 times, then 1 mL of crystal violet staining solution was added to each well, stained for 30 min. PBS washed the cells for several times, and then pictures were taken and counted by Image J analysis.

### 2.15 Migration and invasion testing

A549 and PC9 cells were cultured in six-well plates and treated with siRNAs for 24 h. The Transwell chambers (BD Biosciences, NY, United States) were pretreated or untreated with Matrigel (Corning, NY, USA) to assess the invasive and migratory capacity of the cells. A total of 3*104 cells were seeded into the upper chamber in 200ul of serum-free medium. The lower chamber was supplemented with 700ul of medium containing 10% FBS. The transwell chambers were placed in clean 24-well plates and incubated for 24 h. Migrating or invasive cells were fixed and stained, then photographed under a microscope.

### 2.16 Statistical analysis

Data analysis and image production were done using R software (version 4.1.3) and Graphpad Prism 9. Cell counting and assays were conducted using Image J software. Statistical tests included Wilcoxon and chi-square tests, log-rank test for survival analysis, and Spearman rank correlation for correlation analysis.

## 3 Results

### 3.1 Characterization of lysosome-associated genes

**Figure 1:**
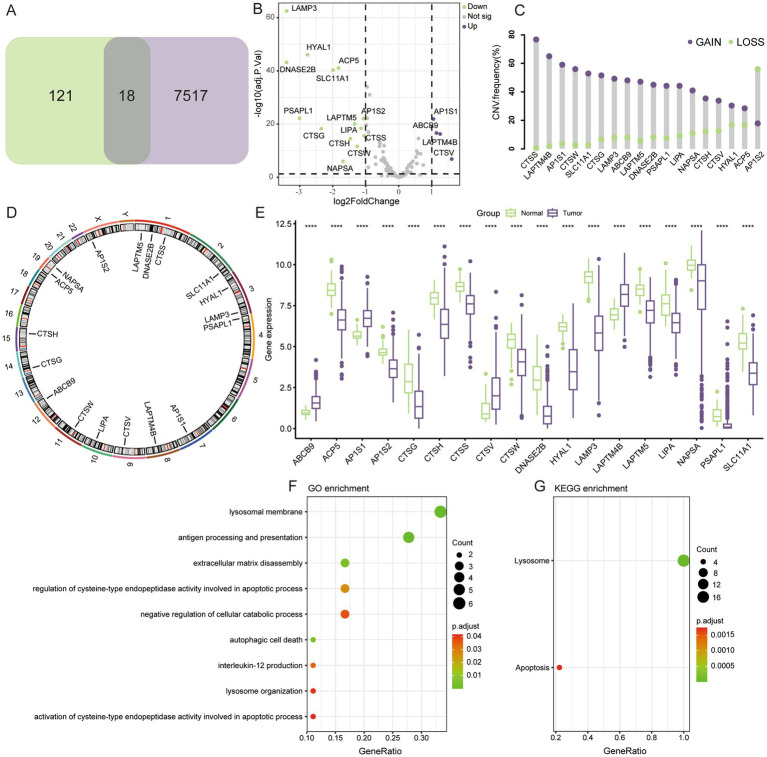
Genomic characteristics of LAGs. **(A)** 18 differentially expressed LAGs were screened. **(B)** Volcano map showing the fold change of LAGs. **(C)** Gain and loss of copy number for 18 key genes. **(D)** Localization of key genes on chromosomes. **(E)** Differential expression of key LAGs in LUAD and normal lung tissue. **(F)** GO and KEGG enrichment analysis of key LAGs confirmed that these genes were related to the structure and function of lysosome. ****p < 0.0001.

By comparing the differences between tumor and normal tissues in TCGA and taking intersections with lysosome-related genes, a total of 18 key LAGs were obtained (Figure 1A), and the volcano plot demonstrates the extent of their differences (Figure 1B). Seventeen of the 18 LAGs had more gene CNV gain than loss, and only AP1S2 had more CNV loss (Figure 1C). The distribution of these 18 genes on different chromosomes was shown in Figure 1D. In terms of expression, only four LAGs were more highly expressed in tumors (Figure 1E), whereas three of the histones that play key degradative roles in lysosomes (CTSG, CTSH, CTSS, and CTSW) were more highly expressed in normal lung tissues, and only CTSV was significantly upregulated in tumors. Analysis by GO and KEGG enrichment revealed that the functions of these genes were mainly focused on the structure of lysosomes, apoptosis, and autophagy (Figures 1F,G).

### 3.2 Construction and validation of LAG risk signature

**Figure 2:**
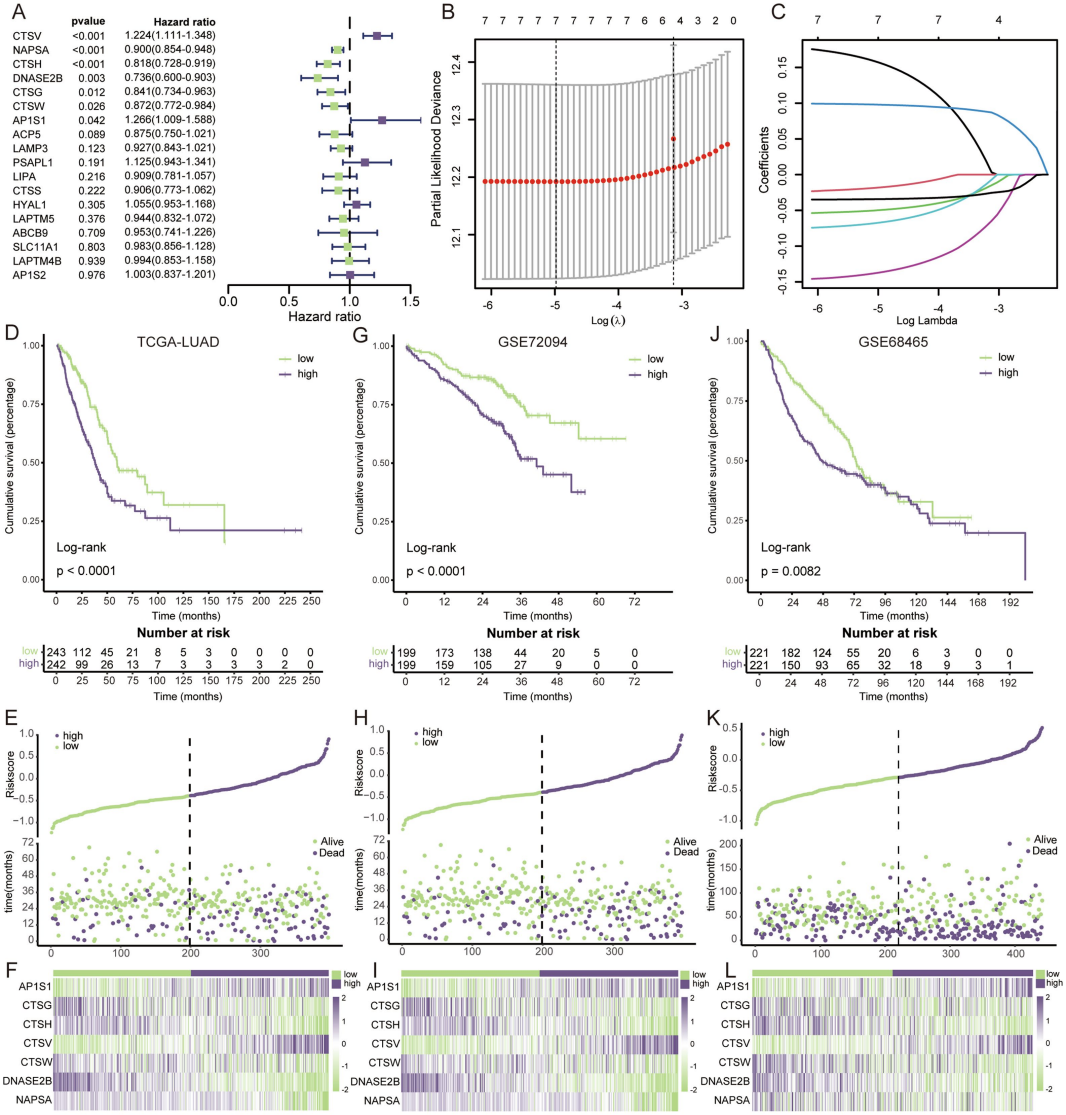
Construction and validation of LAG risk signature. **(A)** Univariate cox regression survival analysis of 18 key LAGs. **(B)** The LASSO Cox regression model was utilized for parameter (lambda) adjustment through 10-fold cross-validation. **(C)** Identification of five key LAGs and their corresponding coefficients. **(D)** KM survival analysis in TCGA dataset. **(E)** The relationship between mortality rates and risk scores in the TCGA dataset. **(F)** The heatmap visualizes the expression patterns within the key LAGs in TCGA dataset. **(G)** KM survival analysis in GSE72094. **(H)** The relationship between mortality rates and risk scores in the GSE72094. **(I)** The heatmap visualizes the expression patterns within the key LAGs in GSE72094. **(J)** KM survival analysis in GSE68465. **(K)** The relationship between mortality rates and risk scores in the GSE68465. **(L)** The heatmap visualizes the expression patterns within the key LAGs in GSE68465.

Univariate cox regression analysis revealed that only 7 of the 18 key LAGs were significantly associated with prognosis (Figure 2A), and these 7 genes were enrolled in the lasso cox regression (Figures 2B,C), and the formula for the LAG risk signature was obtained in the case of optimal lambda value: score = AP1S1*0.15664495-CTSG*0.01797917- CTSH*0.04960209 + CTSV*0.09786986-CTSW*0.06713002-DNASE2B*0.13696602- NAPSA*0.03425495. In the TCGA training set, the LAG risk signature was able to well identify patients with different prognostic status (Figure 2D), and patients in the high-risk group had a higher mortality rate (Figure 2E), and the expression levels of different genes in the two risk groups are shown in Figure 2F. In two validation cohorts, the distributions of KM survival analysis, mortality, and gene expression levels were almost identical to the training set, suggesting that our LAG risk signature had strong predictive performance (Figures 2G–L). In addition, we also analyzed the relationship between different clinical information subgroups of patients and LAG risk signature. The low-risk group has more TNM stages with lower levels (Supplementary Figure S1). The KM curve indicated that LAG risk signature can accurately predict the prognosis of patients without being affected by age, gender, and TNM stage (Supplementary Figure S2).

### 3.3 Construction and validation of the nomogram

**Figure 3:**
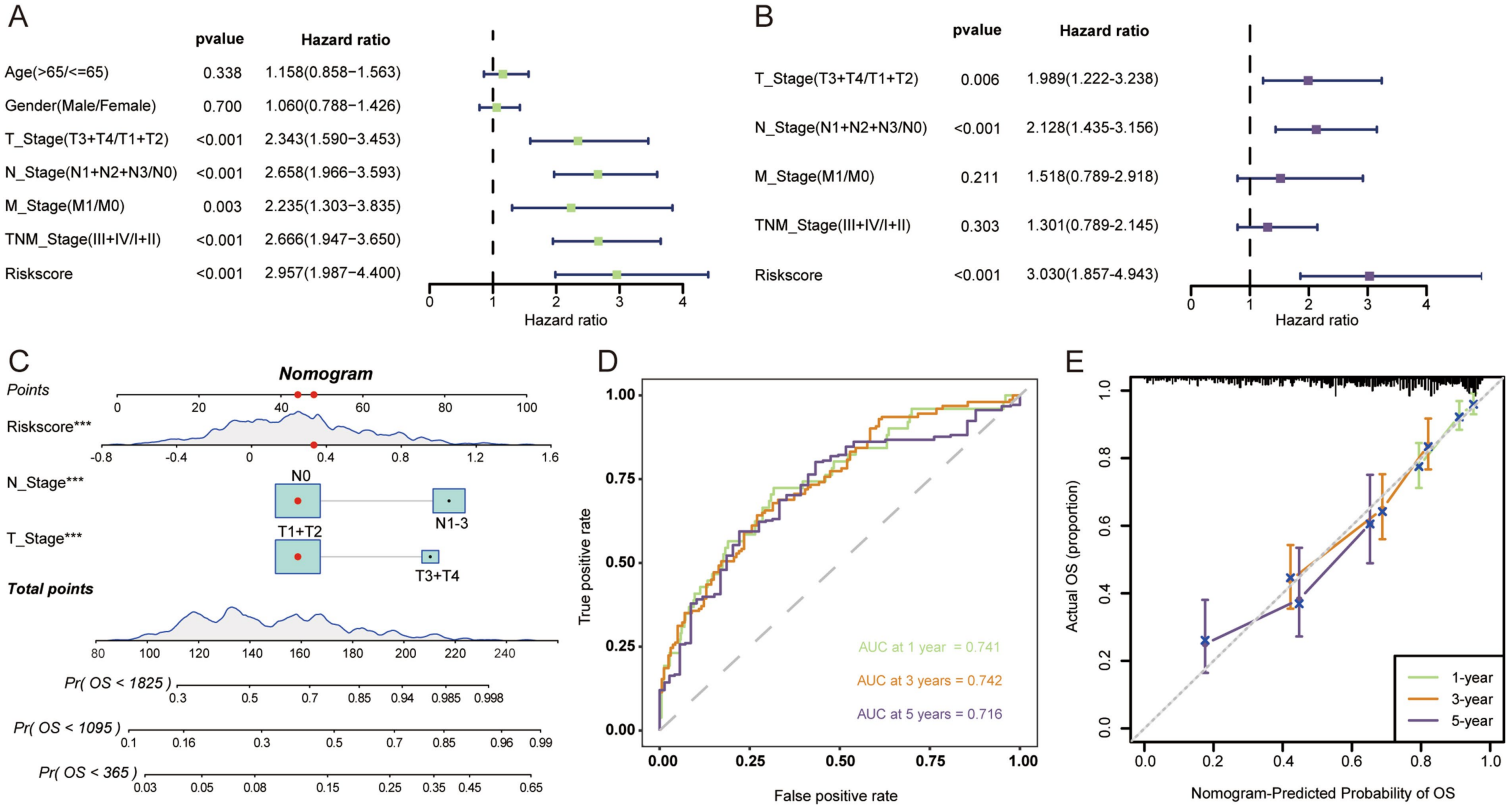
The nomogram was developed to predict the prognosis of LUAD. Univariate **(A)** and multivariate **(B)** Cox regression analysis suggests that risk score is an independent risk factor. **(C)** Constructing a nomogram for quantifying a patient’s likelihood of survival. **(D)** ROC curves were used to evaluate the performance of the nomogram. **(E)** Calibration curves were used to measure the agreement between predicted and true values. ***p < 0.001.

Both univariate and multivariate cox regression analyses confirmed that LAG risk signature was an independent prognostic risk factor (Figures 3A,B). To make a better clinical application scenario for LAG risk signature, we integrated the signature and clinical characteristics and constructed a visualized nomogram prediction model (Figure 3C) 0.1-, 3-, 5-year area under the curve were 0.741, 0.742 and 0.716, respectively (Figure 3D). And the calibration curves also showed the consistency between the nomogram prediction results and the actual results (Figure 3E).

### 3.4 Potential functional pathways between different risk groups

**Figure 4:**
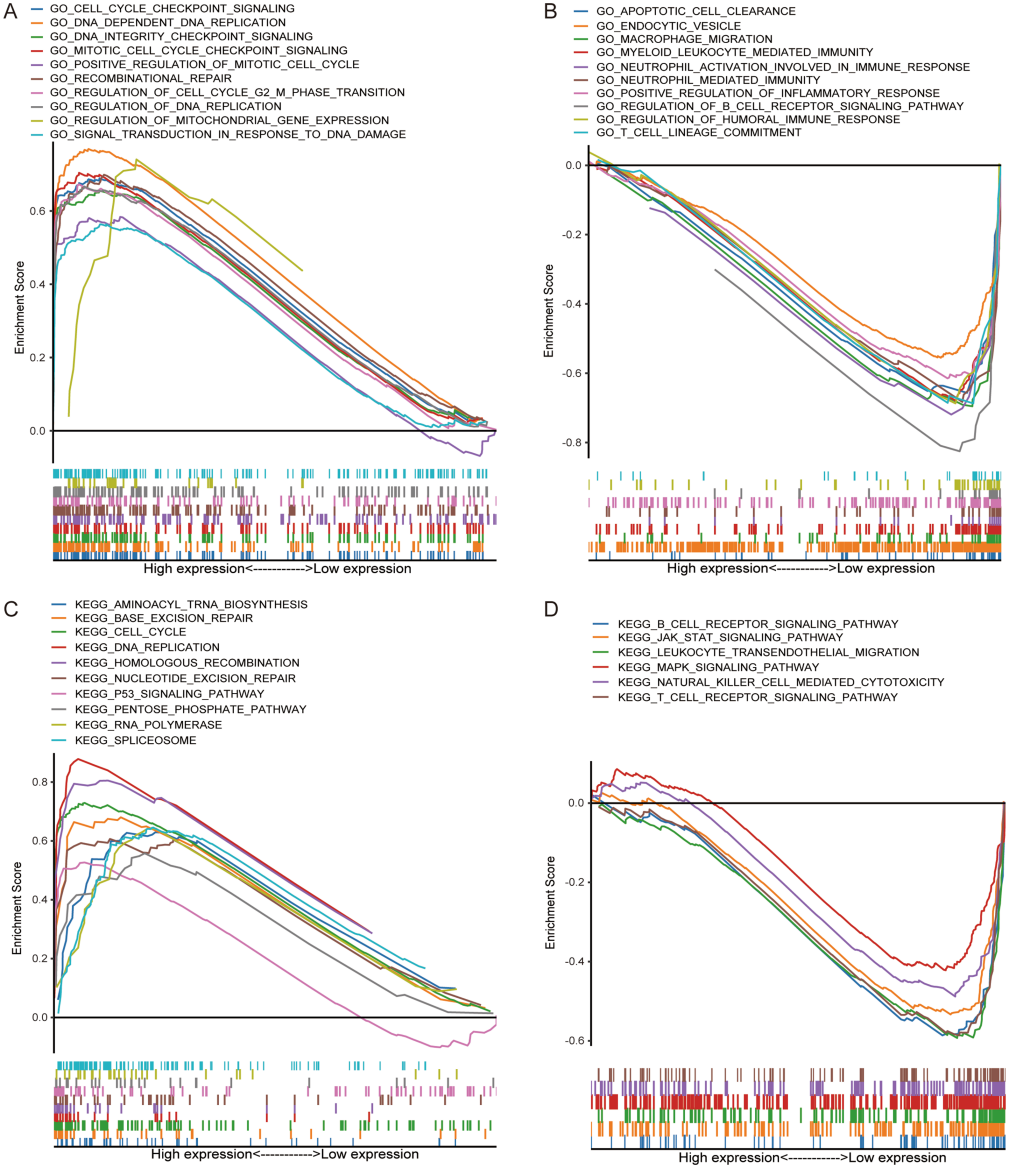
GSEA confirms that the high-risk group was closely related to tumor progression, while the low-risk group was related to immune activation status. GO enrichment in high-risk group **(A)** and low-risk group **(B)**. KEGG enrichment in high-risk group **(C)** and low-risk group **(D)**.

GSEA-GO analysis showed that the high-risk group was mainly involved in the activation of cell cycle signaling, in response to DNA damage (Figure 4A), which suggested that the high-risk group was in a state of continuous cellular proliferation (24), and thus had a worse prognosis. In contrast, extensive activation of the immune status and modulation of the apoptosis was present in the low-risk group (Figure 4B), which apparently inhibited tumor progression and thus improved the prognosis of the patients. The results of GO-KEGG analysis similarly confirmed the above results (Figures 4C,D).

### 3.5 The high-risk group had higher TMB

**Figure 5:**
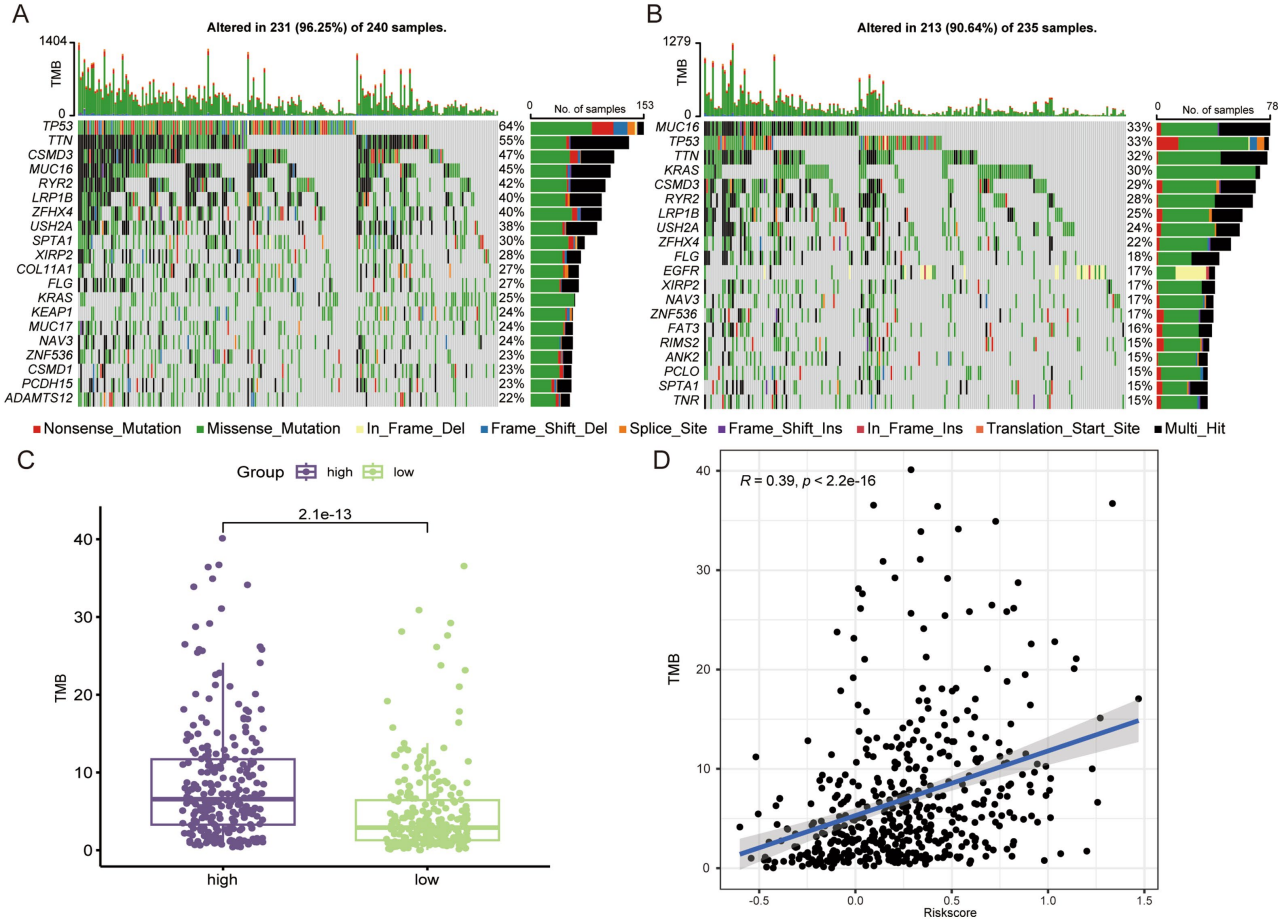
The high-risk group LUAD has higher TMB. Genes with the top 20 mutation frequencies in high-risk group **(A)** and low-risk group **(B)**. **(C)** Comparison of TMB in different risk groups. **(D)** Correlation analysis between risk score and TMB.

Genetic mutations were one of the key factors influencing tumor prognosis (25), and we analyzed TMB in patients from different risk groups. In the high-risk group, the top 5 genes with the highest mutation rates were TP53, TTN, CSMD3, MUC16, and RYR2 (Figure 5A), whereas in the low-risk group they were MUC16, TP53, TTN, KRAS, and CSMD3 (Figure 5B). The incidence of mutation in TP53, which regulates aberrant cell proliferation, between different risk groups also confirmed that the cell proliferation process was more active in the high-risk group. In terms of overall TMB, the high-risk group had a higher tumor mutation load (Figure 5C), and we found a significant positive correlation between risk score and TMB (Figure 5D).

### 3.6 Analysis of the immune microenvironment and prediction of immunotherapy response

**Figure 6:**
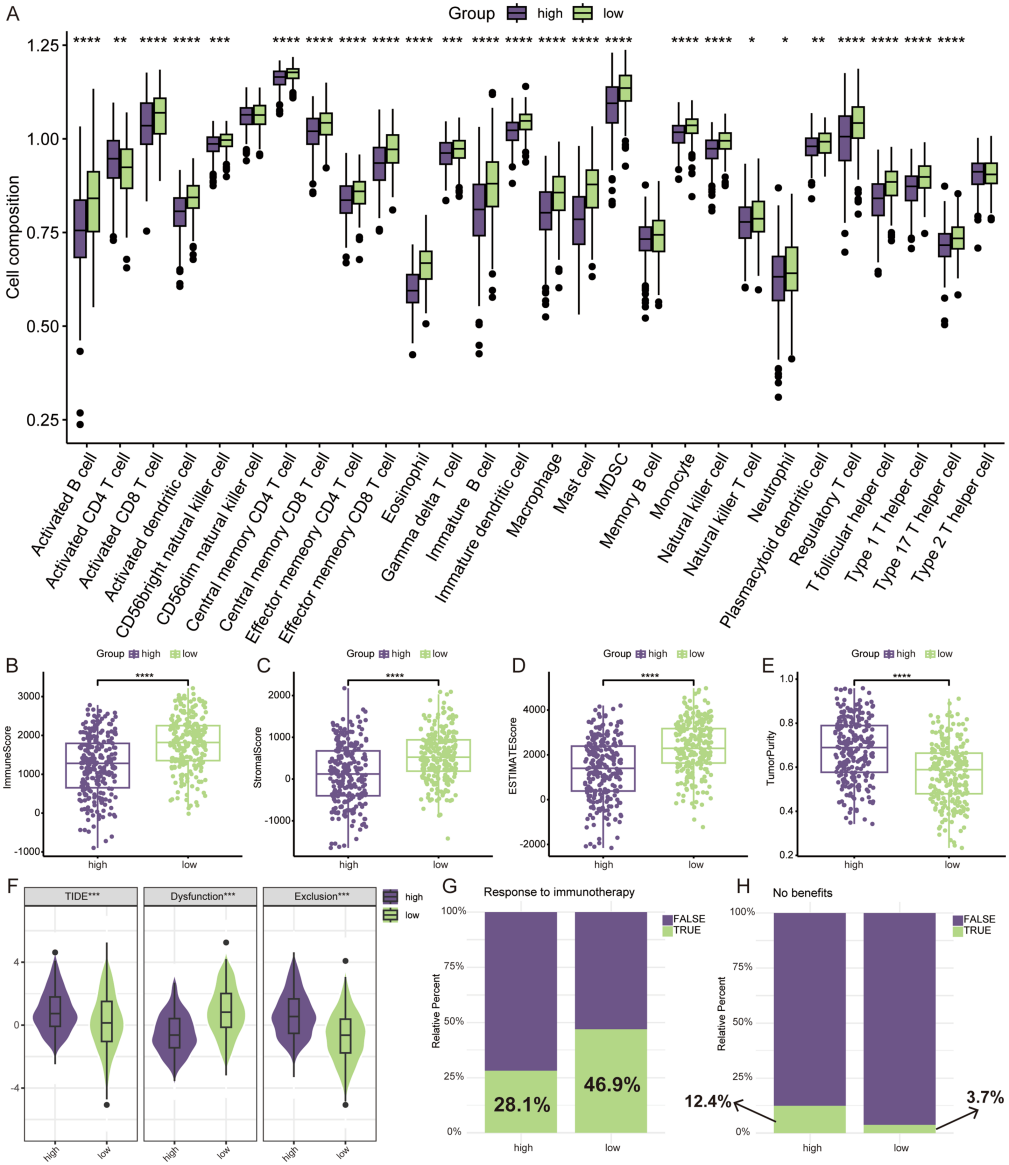
TME assessment and prediction of immunotherapy response. **(A)** Relative infiltration levels of 28 immune cells by ssGSEA. ESTIMATE algorithm for evaluating tumor microenvironment, including immune score **(B)**, stromal score **(C)**, ESTIMATE score **(D)**, and tumor purity **(E)**. **(F)** The difference of TIDE score. **(G)** Prediction of response to immunotherapy through TIDE algorithm. **(H)** No benefits rate to immunotherapy by TIDE algorithm. *p < 0.05, **p < 0.01, ***p < 0.001, ****p < 0.0001.

The results of GSEA suggested that the low-risk group was in a state of immune activation, and we hypothesized that the low-risk group had a richer immune cell infiltration. We first analyzed the extent of immune cell infiltration in the different subgroups. Not surprisingly, there were more abundant immune cells in the low-risk group (Figure 6A). The ESTIMATE algorithm also showed that the low-risk group had higher immune and mesenchymal scores, while the high-risk group had higher tumor purity (Figures 6B–E). We also analyzed the expression differences of some important immune related molecules between different groups, and it is evident that most molecules that promote immune response are highly expressed in the low-risk group (Supplementary Figure S3). The degree of immune cell infiltration largely determines the therapeutic effect of ICIs (26). We used the TIDE algorithm to simulate the prediction of the effect of immunotherapy received by LUAD patients, and the high-risk group had higher TIDE as well as exclusion score (Figure 6F), which implied that they were more likely to be resistant to immunotherapy when receiving ICIs treatment. In the prediction of response to immunotherapy, a significantly higher proportion of the low-risk group responded to immunotherapy than the high-risk group (Figure 6G). Similarly, more people in the high-risk group did not benefit from immunotherapy (Figure 6H).

### 3.7 Characteristics of LAG risk signature in tumor microenvironment

**Figure 7:**
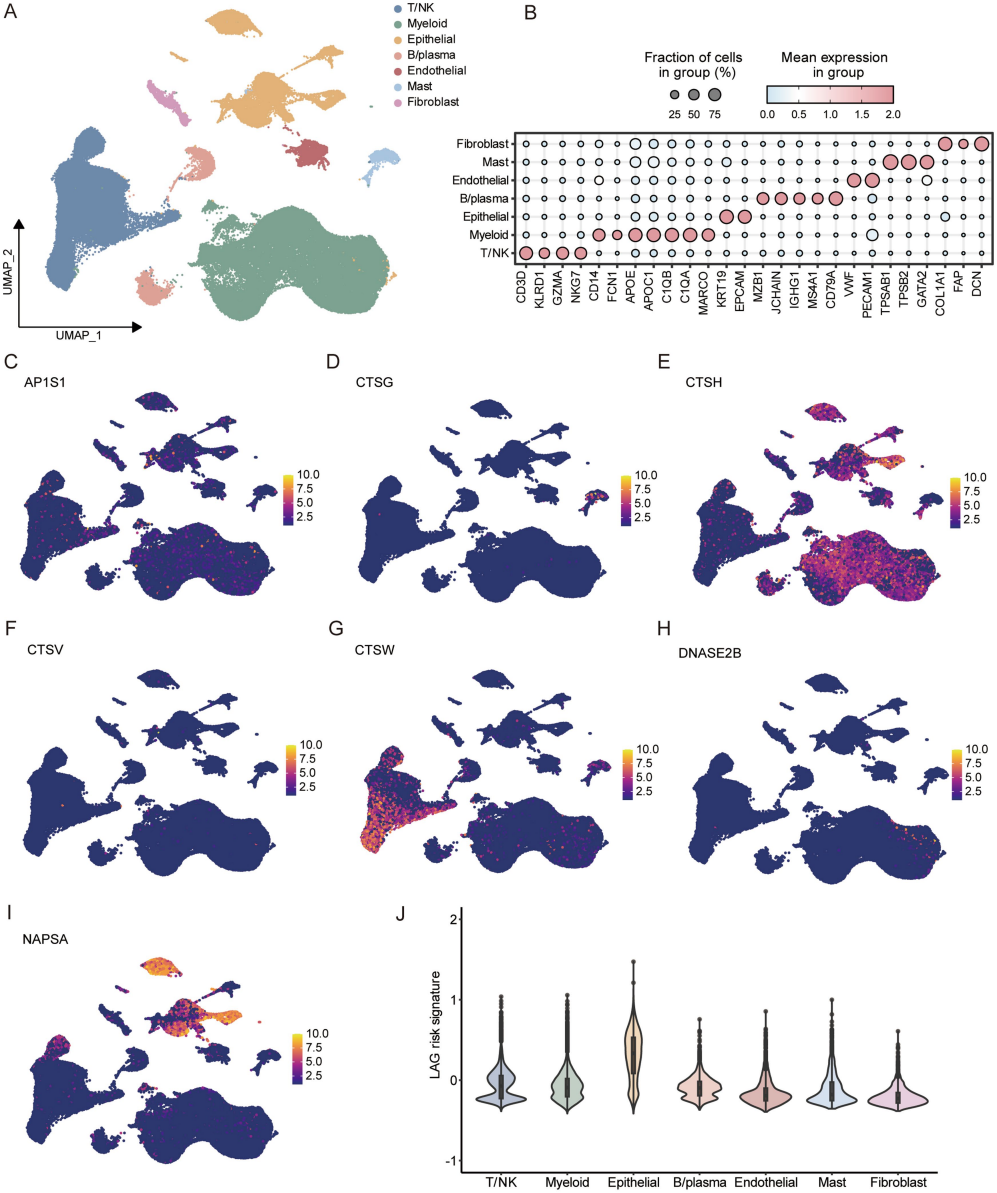
Analysis of tumor microenvironment based on scRNA-seq of LUAD. **(A)** Umap dimensionality reduction maps for 7 cell types. **(B)** Marker for identifying different cell subtypes. Expression of 7 key LAG signature genes at the single-cell level, including AP1S1 **(C)**, CTSG **(D)**, CTSG **(E)**, CTSV **(F)**, CTSW **(G)**, DNASE2B **(H)**, NAPSA **(I)**. **(J)** Comparison of LAG risk scores for different cells.

After data quality control, we obtained a total of 97,019 cells. Based on typical markers, we defined 7 subgroups, including T/NK, myeloid, epithelial, B/plasma, endothelial, mast, and fibroblast (Figures 7A,B). The distribution of all cells in different samples and tissue types was shown in the Supplementary Figure S4, and the umap plot indicated that batch effects have been well removed. Subsequently, we analyzed the expression of seven key genes in the LAG risk signature in the TME. AP1S1, CTSG, CTSV, and DNASE2B were scattered in the TME. CTSH was mainly distributed in epithelial cells and myeloid cells, CTSW was mainly distributed in T/NK cells, and NAPSA was mainly distributed in epithelial cells (Figures 7C–I). Subsequently, we calculated the LAG risk signatures of different cell subpopulations, with epithelial cells having the highest signature (Figure 7J).

### 3.8 Cell communication analysis in different LAG risk groups

**Figure 8:**
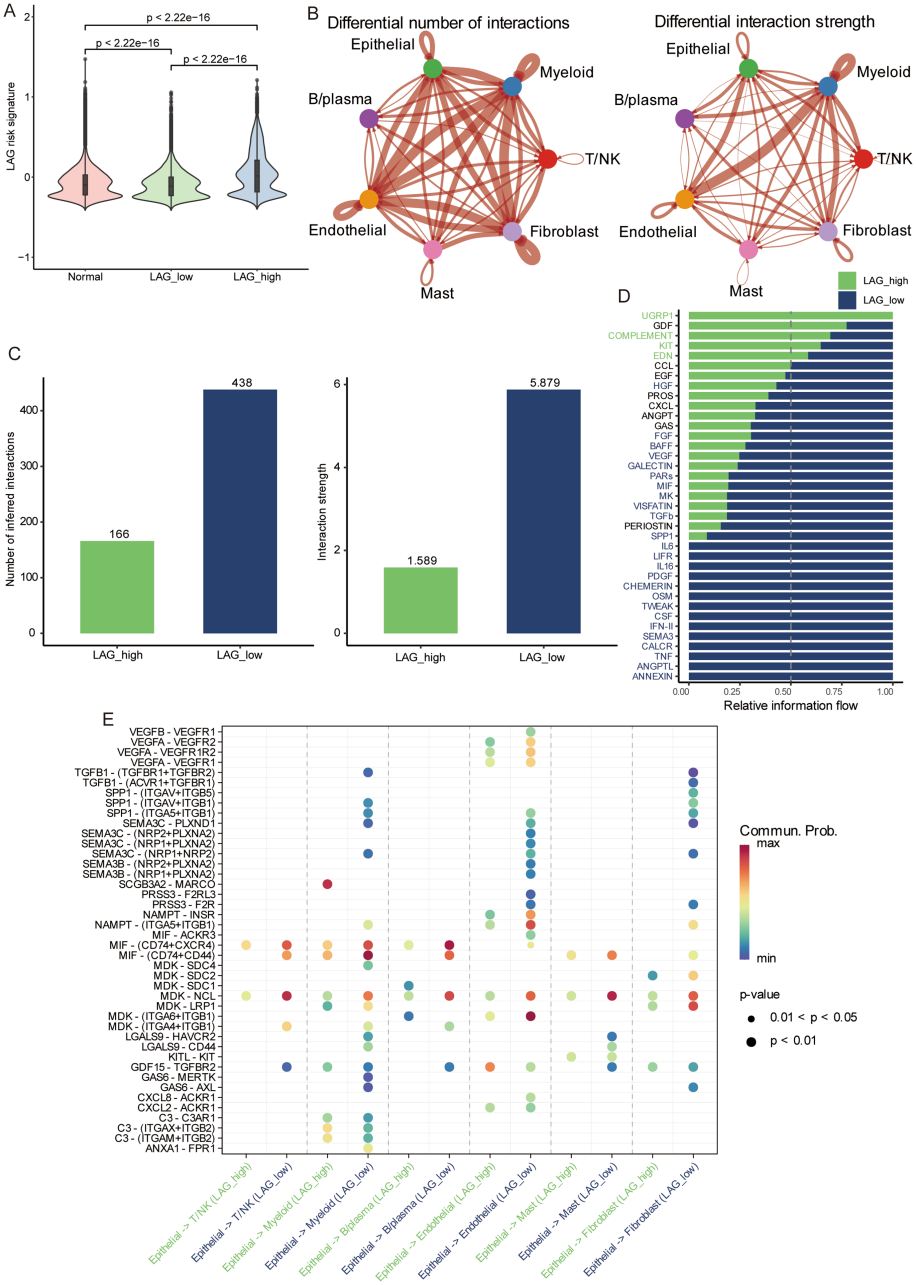
Differences in cellular communication between different risk groups based on scRNA-seq. **(A)** The differences in normal tissue, LAG_low, and LAG_high group. **(B)** The number and strength of cell interactions between different cell types. **(C)** The number and strength of cell interactions in LAG_ low and LAG_ high group. **(D)** Differences of different pathways in LAG_ low and LAG_ high groups. **(E)** Differences and correlations of ligands and receptors in LAG_ low and LAG_ high groups.

We used the median LAG signature score obtained from scRNA-seq of 10 LUAD patients to divide them into LAG high and LAG low groups. LAG_low had the lowest LAG risk signature, even lower than normal tissue (Figure 8A), while a lower LAG risk score meant better prognosis. Then we used CellChat to compare the pathway differences between the LAG_high and LAG_low groups in tumor samples. Overall, compared to the LAG_ high group, the LAG_ low group had a richer intracellular communication network, including both quantity and strength (Figures 8B,C). In terms of specific pathways, the LAG_Low group had a richer activation of inflammatory pathways, such as CXCL, GAS, IL6, IL16, IFN-II and TNF (Figure 8D). Next, we focused on the ligand- receptor differences between epithelial cells and other cell types. It is evident that more ligand receptors were activated in the LAG_Low group, especially in myeloid cells, endothelial cells, and fibroblasts (Figure 8E). We also visualized some pathways between the LAG_high and LAG_low groups, and it was evident that LAG_low group has a richer inflammatory function and chemokine pathway (Supplementary Figure S5), further validating the potential mechanism of better prognosis in the LAG_low group.

### 3.9 CTSH inhibited proliferation and invasion of LUAD

**Figure 9:**
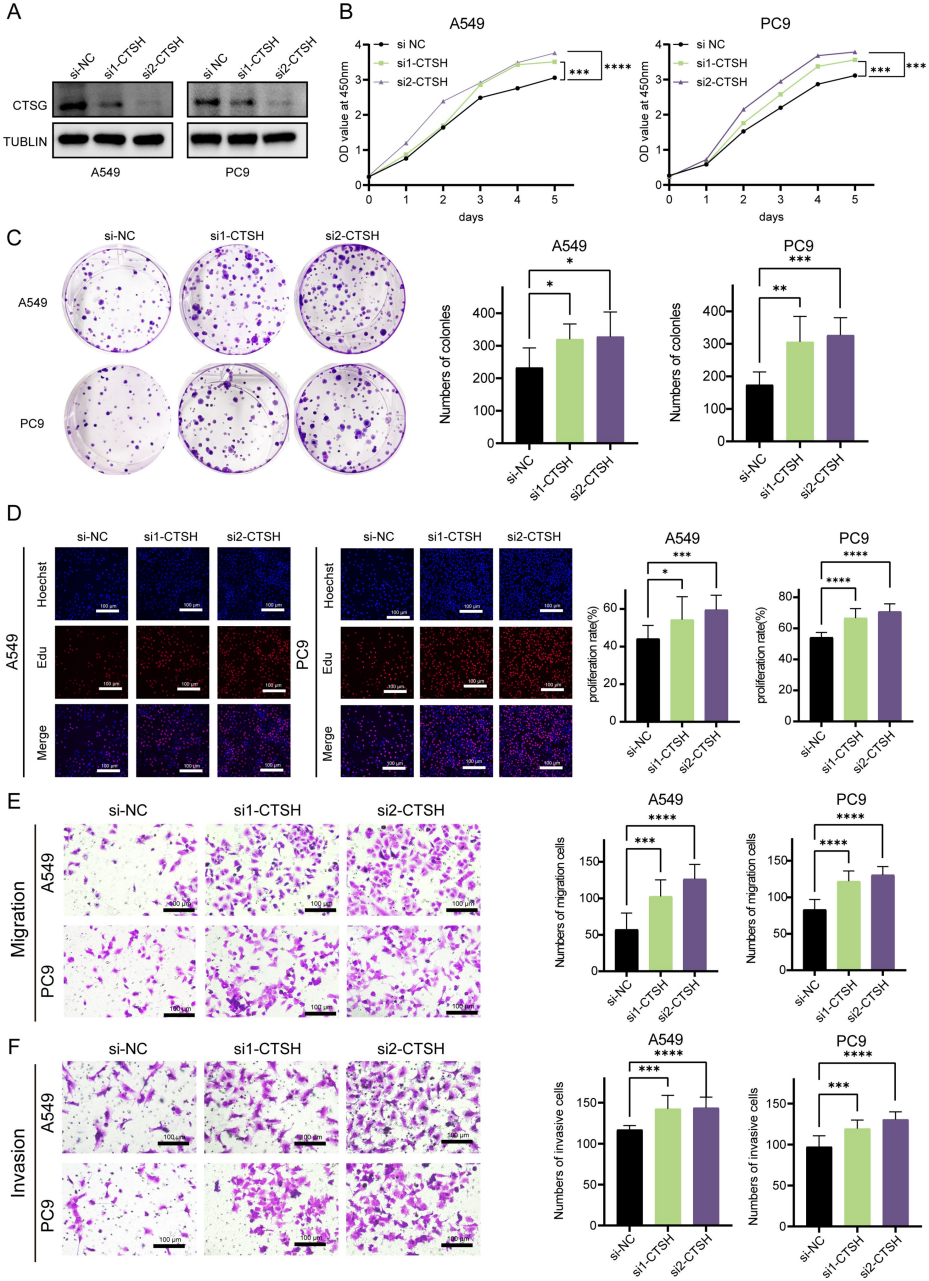
CTSH inhibited the progression LUAD *in vitro* experiments. **(A)** Western blotting demonstrated the knockdown efficiency of CTSH. **(B)** The CCK8 proliferation experiment showed that inhibiting the expression of CTSH enhanced the proliferation of LUAD. **(C)** Plate cloning indicates significant strengthening of clone formation ability after knockdown. **(D)** EDU experiments showed a significant increase in the proportion of LUAD cells in proliferative state after CTSH knockdown. The migration **(E)** and invasion **(F)** ability of LUAD cells increased significantly after CTSH knockdown. *p < 0.05, **p < 0.01, ***p < 0.001, ****p < 0.0001.

Due to the fact that most of the genes composed of LAG signature belong to the cathepsin family, we focus more on the role of proteases. Normally, the cathepsin comes from lysosomes in cells and play an important role in tumors by regulating cell proliferation, autophagy, angiogenesis, invasion, and metastasis (27). Recent studies have found that tissue proteases can also regulate immune responses, especially in tumor-associated macrophages (28). In this study, only CTSH was widely expressed in tumors and myeloid cells, and it may play a more important role in the tumor microenvironment, we further validated the role of CTSH *in vitro*, and bioinformatics analysis showed that it is highly expressed in normal tissues and considered as an oncogene. CTSH was first knocked down in A549 and PC9 (Figure 9A), followed by CCK8 experiments, which showed that knocking down CTSH significantly enhanced cell proliferation in A549 and PC9 (Figure 9B), similarly, the results of colony formation experiment as well as EdU suggested that the proliferation of LUAD was significantly enhanced after knocking down CTSH (Figures 9C,D). In subsequent migration and invasion experiments, knocking down CTSH promoted the progression of lung adenocarcinoma (Figures 9E,F). These results confirm that CTSH plays an important role as a tumor suppressor gene in the development of LUAD.

## 4 Discussion

Lysosomes are the hub of cell survival and cell death, repairing or promoting the death of cells and their organelles in a timely manner in the event of abnormalities, such as injury and senescence, to maintain cellular homeostasis and inhibit tumor development (11). However, in cancer cells, where the demand for energy is greatly increased due to their own proliferation, the process of autophagy carries out self-digestion through lysosomes, which breaks down unnecessary organelles, thus increasing the efficiency of nutrient utilization, and thus sustaining and promoting tumor progression (29). In addition, the lysosome contains a large number of tissue proteases, some of which are involved in the degradation of the extracellular matrix, a key step in the process of tumor metastasis (30). Given the remarkable contribution of lysosomes to the malignant phenotype of tumors, understanding the characterization of lysosomes in cancer progression was important for the development of therapeutic strategies that target aberrantly activated lysosomes (31, 32).

In this study, we constructed the lysosome risk signature using key LAGs, which has superior ability to predict LUAD prognosis and was validated in independent datasets. For increasing the possibility of clinical application, we combined TNM staging and constructed a quantifiable nomogram. The results of GSEA indicated that the high-risk group was in a state of intense proliferation, and we speculated that this phenotype was closely related to cellular senescence, which was an unavoidable process; however, in tumors this process is inhibited, and there is an increase in the expression of cell-cycle checkpoints and lysosomal genes (33), which resists cellular senescence from being cleared. In addition, deletion or mutation of proto-oncogenes or tumor suppressor gene can also cause abnormal changes in lysosomes. TP53 protein can affect the function and stability of lysosomes by regulating the expression of LAGs, influencing the intracellular environment, modulating autophagy processes and regulating the cellular life cycle. Mutations in TP53 may lead to aberrations in these regulatory mechanisms, which in turn affect the degradation and removal of intracellular waste products, thus contributing to tumor progression and therapeutic challenges (34). Our findings revealed that patients in the high-risk group had a disproportionately high rate of TP53 mutations (64% vs. 33%), which clearly affected lysosomal function in tumors.

The LAG risk signature contains seven genes. AP1S1 encoded the shell of a lattice protein, and its down-regulation leads to the degradation of EGFR-containing lysosomes in NSCLC, affecting the intracellular transport of EGFR, which in turn decreases cell-surface levels of EGFR and affects the therapeutic efficacy of EGFR-TKI (35). CTSG, CTSH, CTSW, and CTSV are all cathepsin proteases in lysosomes, and they play different roles in different tumors. The exertion of pro-tumor or anti-tumor effects mainly depends on the differences in tissues and environments (27, 36). Zhu et al. demonstrated that CTSV exerts a pro-metastatic effect *in vitro* and *in vivo*, which may be related to its inhibition of T cell activity (37). CTSW has been implicated in the process of killing T cells and NK cells, but its role in tumors is not well understood (38). It has been reported that CTSH plays a metastasis-promoting role in hepatocellular carcinoma (39), whereas its role in lung cancer is not clear. In our study, we found that it plays a cancer-suppressing role in LUAD, which may be related to the differences in tumor type and microenvironment. CTSG exerts anti-tumor effects in colorectal cancer by negatively regulating the Akt/mTOR/Bcl-2 signaling pathway, and its overexpression promotes apoptosis (40). We analyzed the in vitro functional assay of CTSH and found that it exerts inhibitory effects on the proliferation, migration and invasion of LUAD cells, which suggested that it may be a potential lysosomal-associated target for LUAD, and had certain significance for the development of lysosomal-associated targeted therapies.

Immunotherapy for cancer was an emerging and exciting therapeutic approach, and PD-1/PD-L1-based immune checkpoint inhibitors have demonstrated superior therapeutic efficacy in a variety of solid tumors (41). However, due to lack of immunogenicity, insufficient cytotoxic T-cell infiltration, and acquired resistance, the vast majority of patients do not respond to ICIs (42, 43). Our study analyzed the components of the immune microenvironment by multiple methods and found that patients in the low-risk group were in a state of immune activation with a higher degree of immune cell infiltration, and thus had better treatment response in the prediction of treatment response to ICIs (46.9% vs. 28.1%) (20).

There are some limitations of our study. First, RNA-seq was derived from public databases, and further prospective clinical trials are needed to obtain real-world data. Second, the potential link between lysosomal risk signature and the immune microenvironment as well as immunotherapeutic response needs further experimental exploration. Finally, further animal experiments are still needed to verify that CTSH exerts an oncogenic role in LUAD.

## 5 Conclusion

In conclusion, we developed a lysosomal risk signature, which is a reliable predictor of prognosis in LUAD patients and evaluates the efficacy of treatment with ICIs in LUAD patients. In addition, CTSH inhibits the progression of LUAD and is expected to be a new target for LUAD treatment.

## Data Availability

The datasets presented in this study can be found in online repositories. The names of the repository/repositories and accession number(s) can be found at: https://www.ncbi.nlm.nih.gov/geo/, GSE72094; https://www.ncbi.nlm.nih.gov/geo/, GSE68465; https://www.ncbi.nlm.nih.gov/, TCGA-LUAD.
