# Supplementary figures and images for: The lysosome-related characteristics affects the prognosis and tumor microenvironment of lung adenocarcinoma
Source: Front Med (Lausanne). 2025 Jan 7;11:1497312. doi: 10.3389/fmed.2024.1497312 (PMC11746080; doi:10.3389/fmed.2024.1497312)

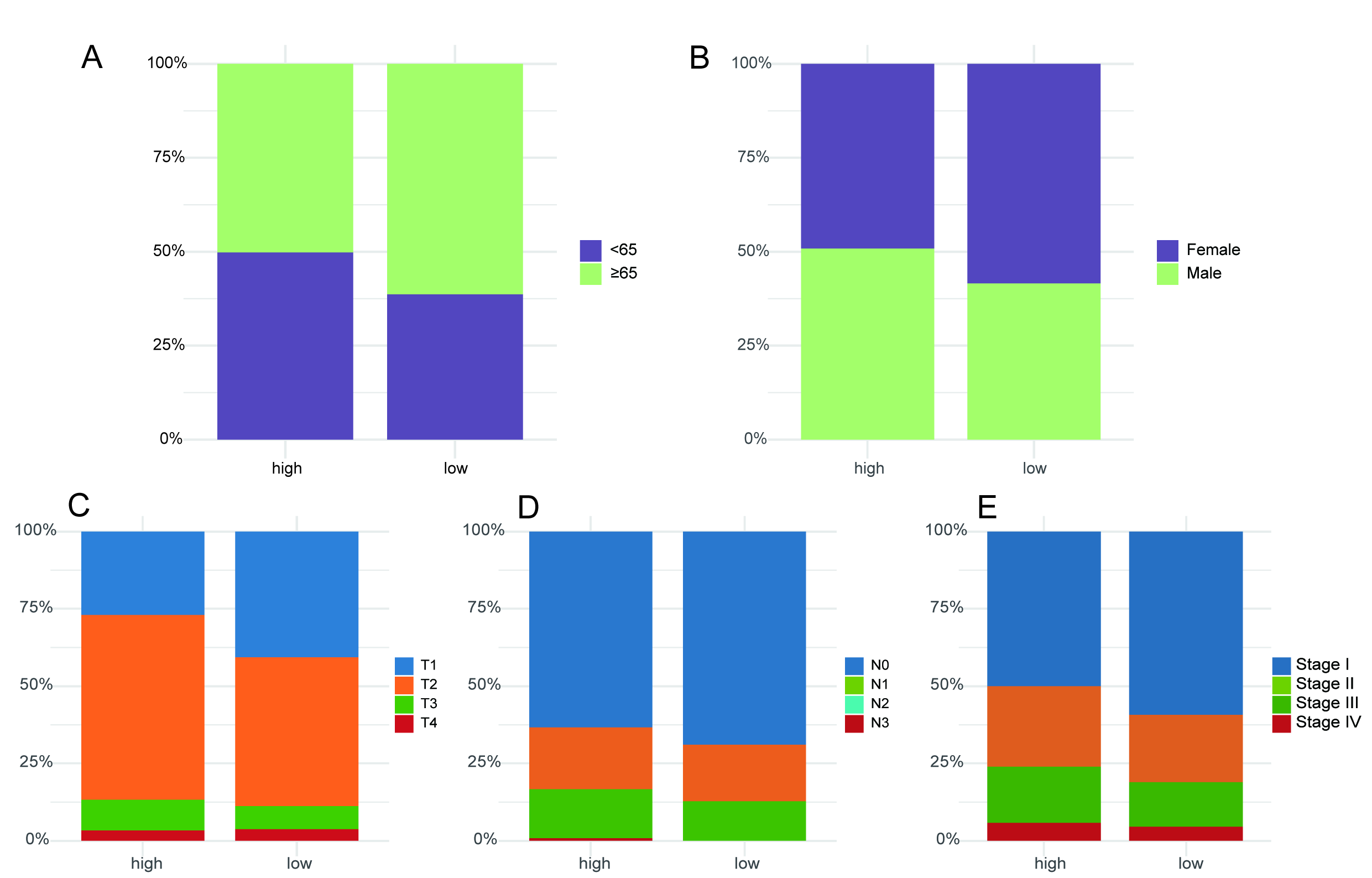

Supplement: Supplementary file 1 [file Image_1.TIF]

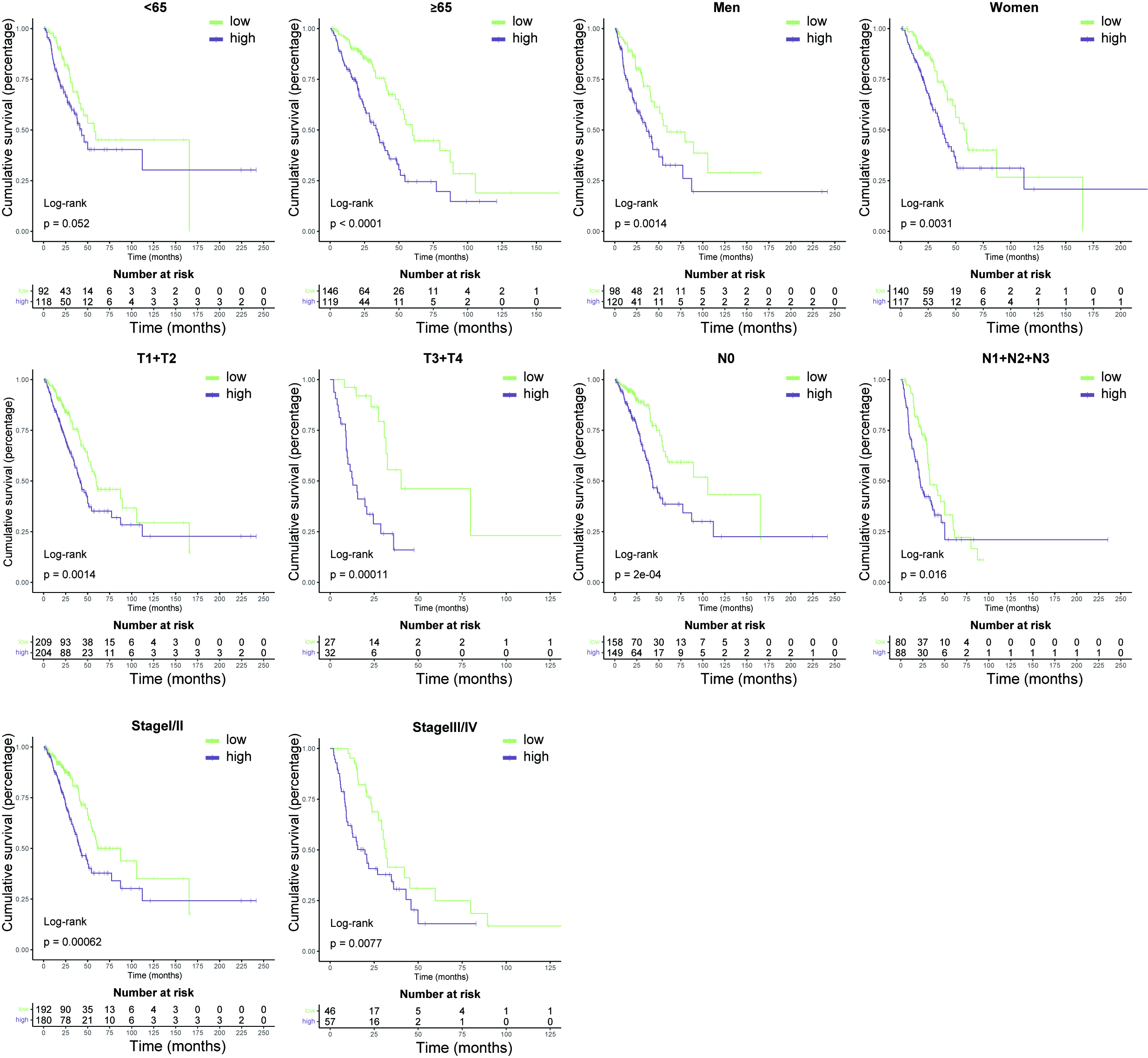

Supplement: Supplementary file 2 [file Image_2.TIF]

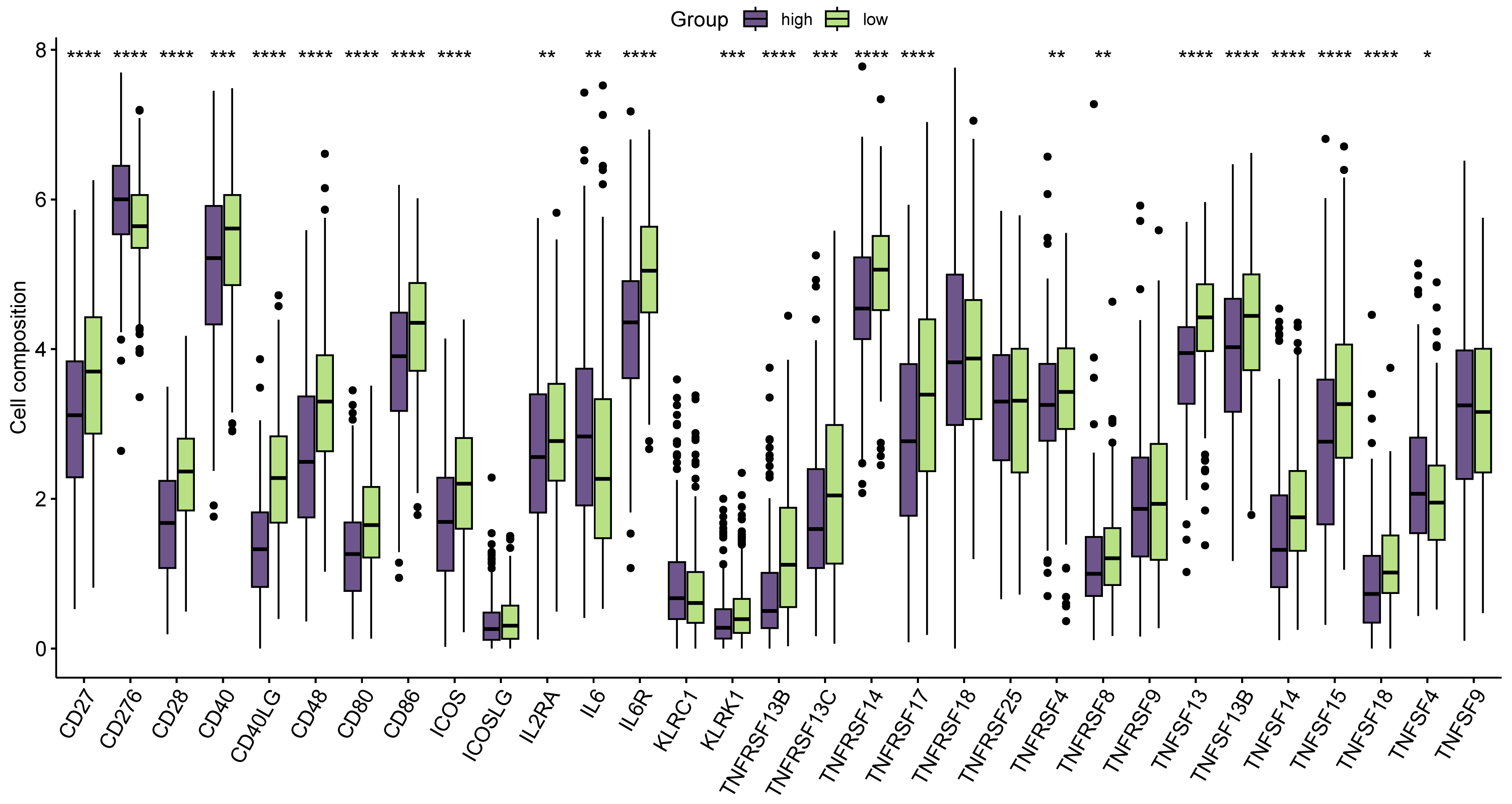

Supplement: Supplementary file 3 [file Image_3.TIF]

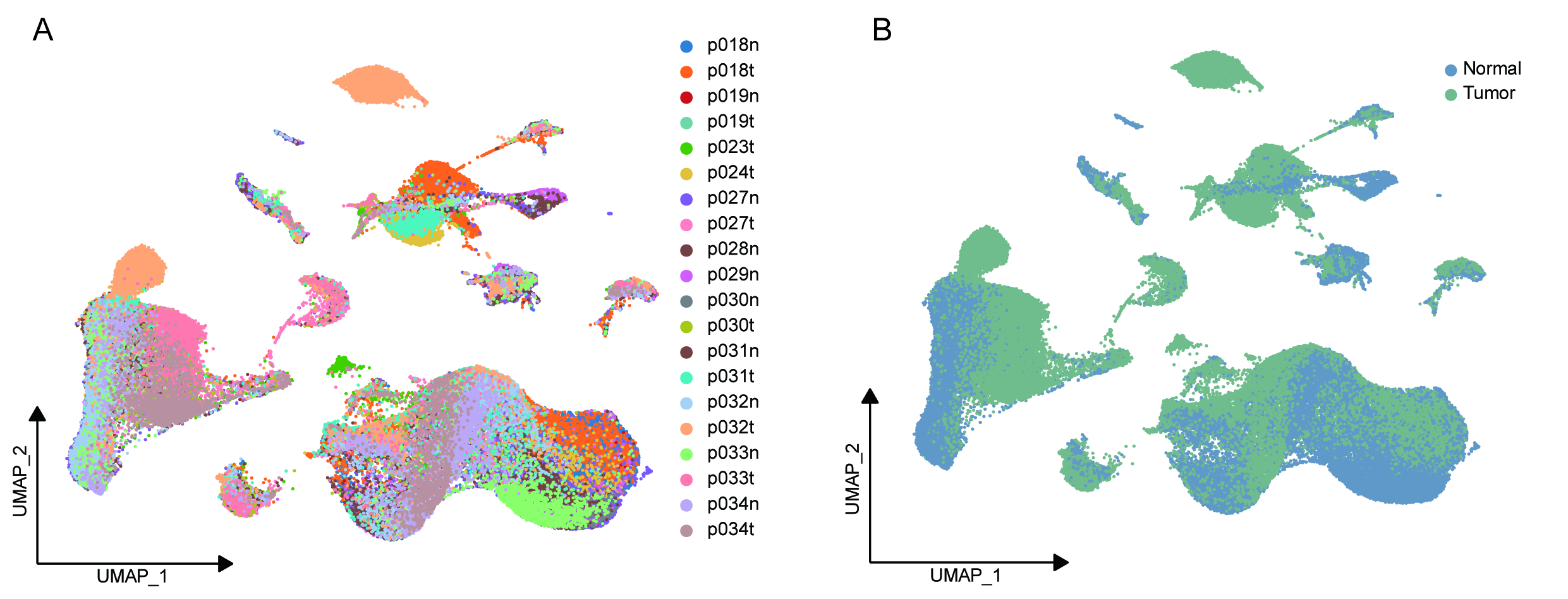

Supplement: Supplementary file 4 [file Image_4.TIF]

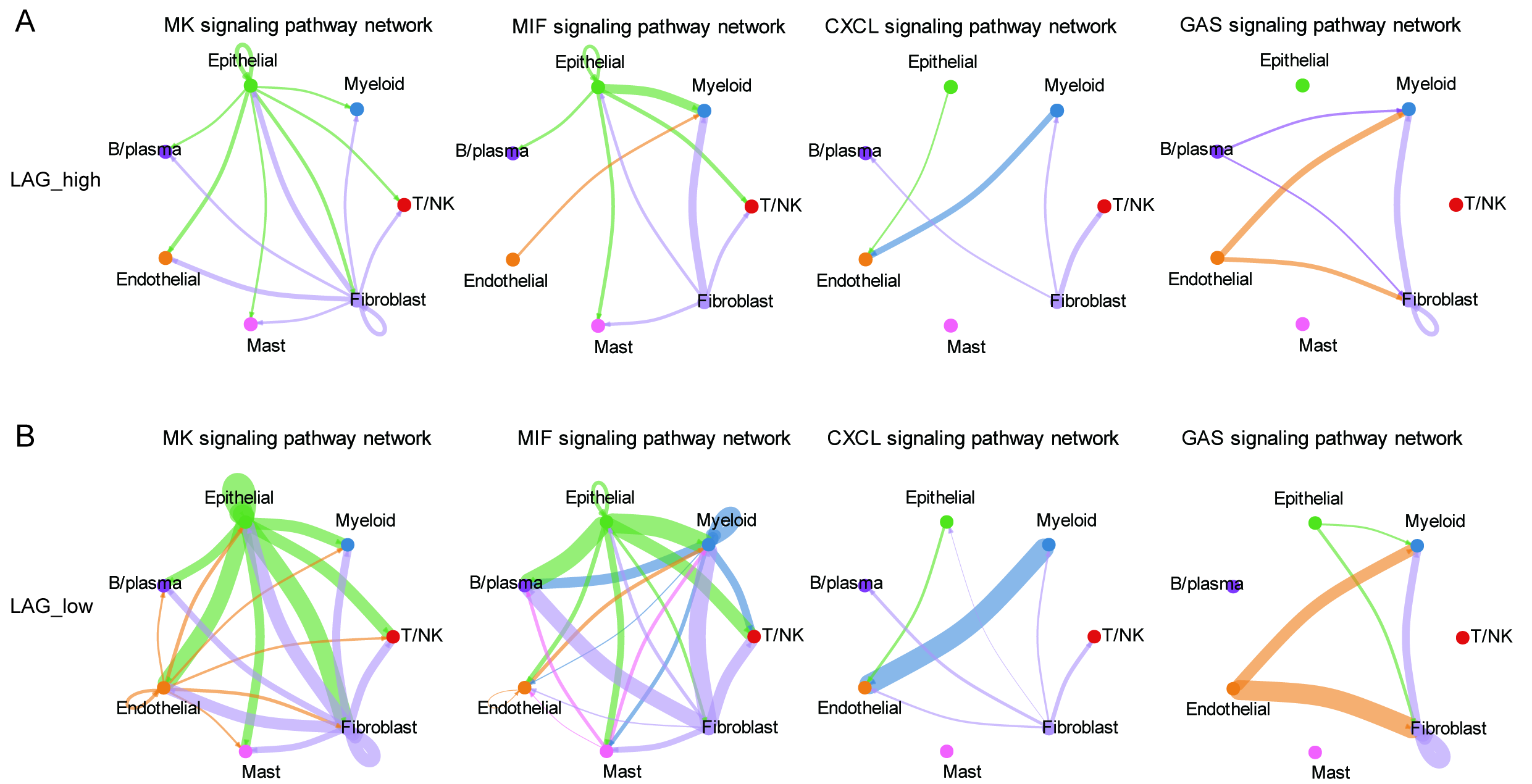

Supplement: Supplementary file 5 [file Image_5.TIF]
